# Neovascular glaucoma in a pediatric patient with neurofibromatosis type 1: a case report

**DOI:** 10.1186/s12886-020-01438-5

**Published:** 2020-04-28

**Authors:** Sha Liu, Li Ran, Dongmei Qi, Xiaohong Meng, Tao Yu

**Affiliations:** Department of ophthalmology, the first hospital affiliated to Army Medical University (Southwest Hospital), Chongqing, 400038 China

**Keywords:** Neovascular glaucoma, Retinal vasoproliferative tumors, Neurofibromatosis type 1, Pediatric

## Abstract

**Background:**

To report a case of a young patient with neurofibromatosis type 1 (NF1).

Methods: Here we review the treatment administered to a 7-year-old NF1 patient with neovascular glaucoma as the primary diagnosis.

**Case presentation:**

A 7-year-old boy developed visual loss in the right eye associated with periocular pain and ipsilateral headache that had persisted for 1 week. The patient’s condition did not improve after treatment with topical or systemic glaucoma medications. Fundus examination of the right eye showed superotemporal retinal vasoproliferative tumors (RVPT). Near-infrared reflectance scans of the left eye’s fundus revealed bright patchy regions, scattered across the posterior pole; systemic examination showed café-au-lait spots all over the patient’s body. The patient had a clear family history. Genetic testing confirmed NF1. The right eye was treated with intravitreal ranibizumab injection, retinal lesion cryotherapy, and transscleral ciliary body photocoagulation. After treatment, RVPT scarring was observed. The patient’s intraocular pressure remained within normal limits.

**Conclusions:**

We report a rare case of neurofibromatosis in a pediatric patient with neovascular glaucoma accompanied by RVPT. We suggest that evaluations of young patients with neovascular glaucoma should include careful attention to the overall condition of the patient and his/her parents, as well as family history. If necessary, NF1 molecular testing should be performed to avoid a missed diagnosis or misdiagnosis.

## Background

Neurofibromatosis type 1 (NF1) is an autosomal dominant genetic disease, the clinical manifestations of which are diverse, often involving the nervous system, skin, eyes, bones, and other organs. The ocular manifestations of NF1 [1] have been reported to include iris Lisch nodules, optic glioma, retinal abnormalities, congenital glaucoma and so on. In children, neovascular glaucoma is rare, which is more commonly seen in diseases associated with angiogenic neovascularization, such as retinoblastoma, Coats disease, retinopathy of prematurity, primary hyperplastic persistent vitreous and chronic uveitis [2]. To our knowledge, neovascular glaucoma in NF1, accompanied by retinal vasoproliferative tumor (RVPT) has rarely been reported. Here we report a case of NF1 with RVPT, which resulted in neovascular glaucoma in a pediatric patient. Below we describe the clinical findings and outcomes of the case.

## Case presentation

**Fig. 1:**
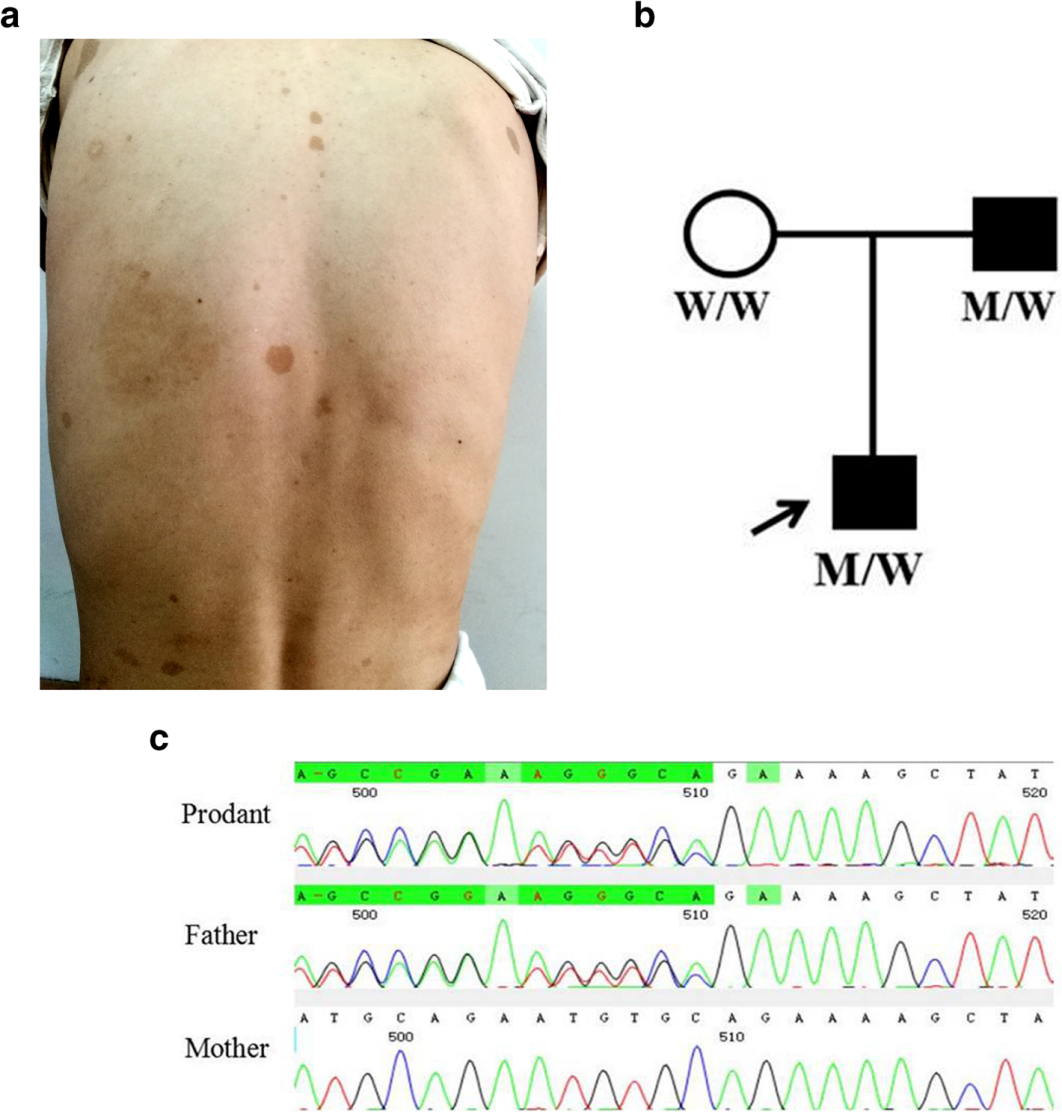
The skin, pedigree and sequence chromatography of the patient. **a**. The photo of the patient’s trunk, showing irregular café-au-lait spots; **b**.. Pedigree. W: wild, M:c.738del A. **c**. One novel sequence change detected in the proband and father with NF1 is shown (right column) compared with corresponding normal sequences (mother)

A 7-year-old boy presented with right eye (RE) pain, decreased vision, and ipsilateral headache for 1 week. The ophthalmologic examination revealed that visual acuity (VA) was light perception in his RE and 1.0 in the left eye (LE); RE conjunctival hyperemia, corneal edema, iris neovascularization, ectropion uveae and 8 mm pupil were seen; posterior segment opacity. The intraocular pressure (IOP) was 59 mmHg in RE and 15 mmHg in LE. There were no obvious abnormalities in LE. B-ultrasonography revealed haziness in the vitreous of RE, with no retinal detachment. The child was otherwise healthy and born at full term. The physical examination revealed that the skin of the trunk was scattered with irregular café-au-lait spots (> 6) of 0.5–5 cm in diameter (Fig. 1a). The brain MRI has been performed and the images show there is no presence of optic glioma and other NF1-related change.

Physical examination of the parents revealed numerous irregular café-au-lait spots on the father’s trunk. Examination of the father’s eyes showed that his best corrected VA was 1.0 with no obvious abnormality. Near-infrared reflectance (NIR) revealed bright patchy regions scattering across the fundus.

**Fig. 2:**
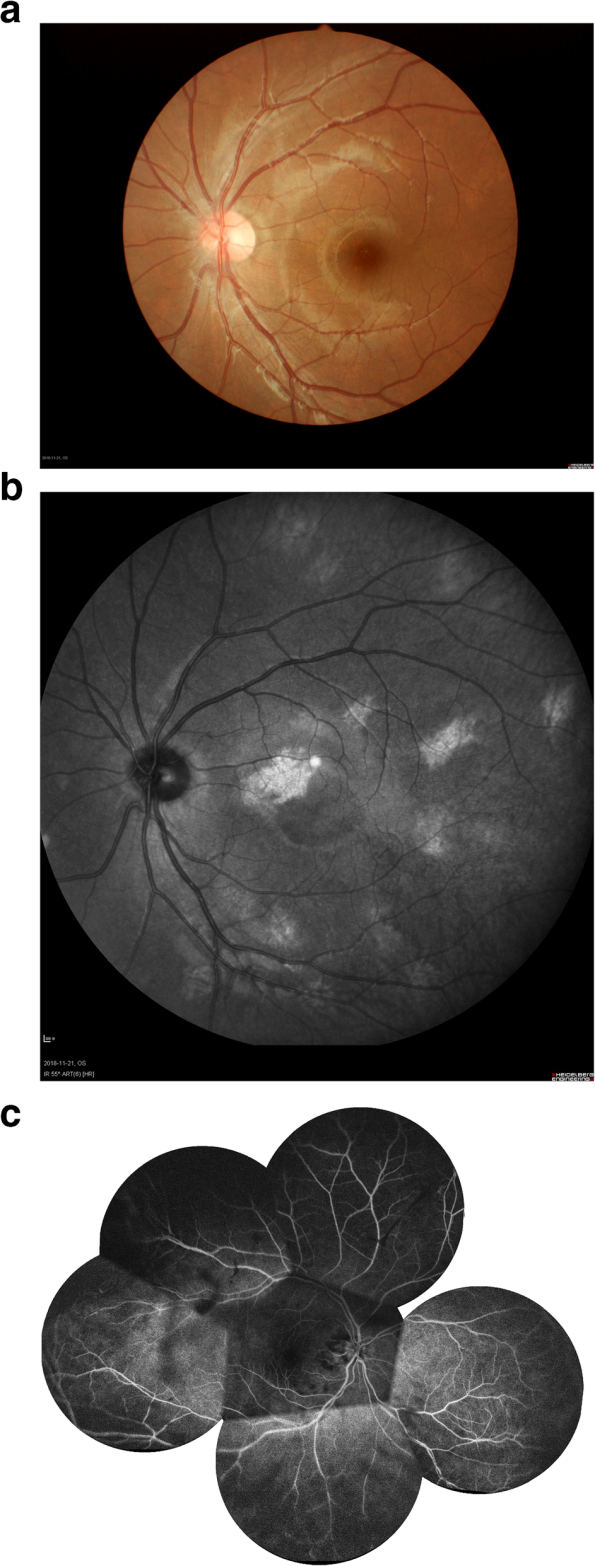
The patient’s left eye fundus and fluorescein angiography. **a**. The color photo of the patient’s left eye shows no abnormalities; **b**. The NIR of the patient’s left eye shows multiple lesions appearing as bright, patchy regions in the posterior poles. **c**. Fluorescein angiography of the left eye showed that the superotemporal retina had a large area of low fluorescence and no abnormal fluorescence was observed

**Fig. 3:**
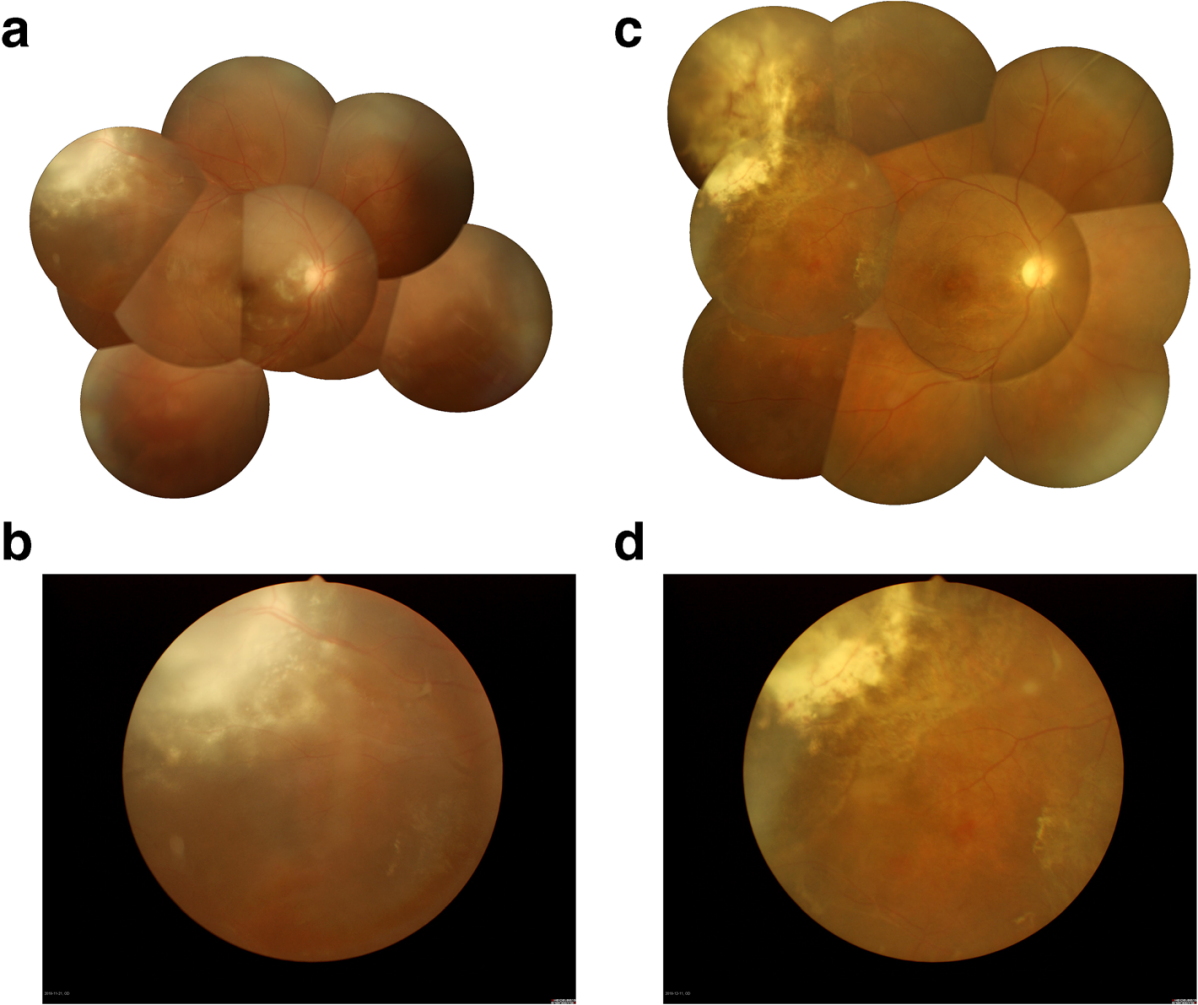
Changes to retinal lesions in the patient’s right eye before and after cryotherapy. **a**. A color fundus photograph obtained before treatment was administered shows a nodular change in superotemporal retina, gray-white in color, with an unclear boundary; **b**. A magnified portion of the photograph presented in 4a, showing blood vessels around the lesion; **c**. A color fundus photograph obtained after treatment shows scar formation in the area of the superotemporal lesion, with a clear boundary; **d**. A magnified portion of the photograph presented in 4c, showing the disappearance of abnormal blood vessels from the area surrounding the lesion

After the admission of the patient, the IOP remained over 40 mmHg for 2 days even with intravenous Mannitol 250 ml twice a day, Acetazolamide 250 mg twice a day and Timolol 0.5% twice a day to lower his IOP. Because of the failure of medical therapy, the third day after admission, intravitreal ranibizumab was injected in the patient’s RE. The next day, IOP decreased to 16 mmHg; VA was hand motion; corneal edema had resolved; iris neovascularisation disappeared. Fudus findings were unclear margin of the optic disc and abundant yellow-white exudates which were observed at the superotemporal retinal nodular bulge; the vessels could be seen at the margin of the nodule (Fig. 3a, b). These findings suggested a diagnosis of RVPT. The fundus of LE was normal (Fig. 2a). Fluorescein angiography of the fundus showed that the superotemporal retina had a large area of low fluorescence and no abnormal fluorescence was observed in LE (Fig. 2c). NIR revealed bright, patchy regions scattering across the posterior pole of LE (Fig. 2b).

Retinal lesion cryotherapy was performed in RE at 7 days after admission and IOP was still high as about 40 mmHg. Transscleral ciliary body photocoagulation was therefore performed. After the procedure, IOP decreased to 20 mmHg. The superotemporal retina nodular lesion was flattened, and the amount of exudates was significantly decreased. The newly formed blood vessels were occluded, and scars could be seen surrounding the lesion (Fig. 3c, d). The patient’s condition is stable till the last follow-up.

DNA samples were taken from the family members and tested by NGS and Sanger sequencing verification and revealed that the patient and his father had a novel mutation in the NF1 gene (Fig. 1b, c). This confirmed a final diagnosis of NF1.

## Discussion and conclusions

The formal diagnostic criteria for NF1 were first formulated by the National Institutes of Health Consensus Development Conference in 1987 [3]. The diagnosis of NF1 is now made if a patient has any two of seven listed clinical characteristics summarized by Ferner in 2007 [4]. According to the NF1 diagnostic criteria, the clinical characteristics of this case are consistent with the first criterion (≥ 6 café-au-lait spots) and with the seventh criterion (one of the immediate family members wasaffected). Based on these findings, a clinical diagnosis of NF1 was established. In recent years, some scholars have found that NIR fundus images reveal characteristic changes in NF1 patients. These patients tend to have bright, patchy regions in the posterior pole, with incidence up to 97.5% (second only to café-au-lait spots, 98%) [5]. Some pathological studies on NF1 patients [6] also report this change, as well as observations of neural crest-derived melanocytes and nerve cell proliferation in the choroid, which thickens the fundus of the posterior and mid-peripheral regions. Melanocytes are rich in melanin, and the melanin absorbs near-infrared light back-scatter, which appears as bright, patchy regions, scattered across the posterior pole. Some scholars^5^ therefore believe that this change in NIR can be used as a diagnostic criterion for NF1. The NIR findings for our patient and his father both showed this change, which confirmed the clinical diagnosis of NF1.

Common NF1 eye manifestations include congenital glaucoma with ipsilateral upper eyelid plexiform neurofibroma and ectropion uveae or iris Lisch nodules [7]. In this case, at the time of the initial diagnosis, neovascular glaucoma observed, with iris neovascularization and ectropion uveae, but no iris Lisch nodules, eyelid neurofibroma, or optic glioma. The incidence of neovascular glaucoma in children is very rare; according to reports, NF1 is one possible cause [8]. Studies have shown that mutations in the NF1 gene may lead to the hyperproliferation of perivascular cells and endothelial cells, thereby enhancing the retinal response to ischemia and causing RVPT. RVPT is not a true tumor, but, rather, a reactive lesion of glial cells and blood vessels [9]. The growth of RVPT is driven by elevated levels of vascular endothelial growth factor (VEGF). Some authors report that RVPT secrete VEGF [8]. VEGF plays an important role in the development of new blood vessels. In this case, the patient with NF1 was diagnosed with neovascular glaucoma accompanied by RVPT. After the intravitreal injection of an anti-VEGF agent, in addition with retinal lesion cryotherapy and transscleral ciliary body photocoagulation, the patient’s IOP returned to normal, with the RVPT scarring.

Cases of NF1 that begin with neovascular glaucoma accompanied by RVPT are quite rare. So far, only 3 cases have been reported in the literature [8]. We compared characteristics of those three reported cases with those of the patient from the present study. These findings show our case has no systematic involvement, but has genetic evidence and positive family history. Therefore, our diagnosis would be more convincing.

Based on the findings presented above, we suggest that evaluations of young patients with neovascular glaucoma should include careful attention to the overall condition of the patient and his/her parents, as well as family history. If necessary, NF1 molecular testing should be performed to avoid a missed diagnosis or misdiagnosis. In the case we first report one novel mutation in NF1 in association Chinese patients with NF1 without systemic findings, and the result expands the mutation spectrum of NF1.

## Data Availability

All data are shown in the figures.

## Abbreviations

NF1: Neurofibromatosis type 1
RVPT: Retinal vasoproliferative tumor
RE: Right eye
VA: Visual acuity
LE: Left eye
IOP: Intraocular pressure
NIR: Near-infrared reflectance
VEGF: Vascular endothelial growth factor
